# GMO approvals under pressure: How climate shocks shape policy across nations

**DOI:** 10.1371/journal.pone.0332298

**Published:** 2025-10-22

**Authors:** Xiangwen Kong

**Affiliations:** Department of Agricultural Economics and Rural Sociology, Auburn University, Auburn, Alabama, United States of America; Federal University Otuoke, NIGERIA

## Abstract

While GMO cultivation is often viewed as a strategy to buffer agriculture against climate change, its regulation remains controversial across countries. This study explores how climate shocks influence global patterns of genetically modified organism (GMO) approval. We develop a theoretical model and predict that climate shocks reduce the likelihood of GMO approvals in countries lacking a comparative advantage in GMO production. To test this, a local projection method is used to estimate the cumulative effects of climate shocks on approval activity. The results show that climate shocks tend to delay approvals, especially in countries with low GMO development capacity. These findings suggest that climate change may amplify regulatory inertia and intensify global policy divides on GMOs, raising concerns about the adaptability of global food systems under increasing environmental stress.

## 1. Introduction

Climate shocks—such as droughts, heat waves, cold spells, floods, and storms—pose significant threats to agricultural production and food security by driving up prices and increasing the risk of hunger. A substantial body of literature has established a causal link between climate shocks and reductions in crop yields; for instance, without adaptation, climate change is projected to lower wheat yields in North America by 1.0–10.0% per degree of warming [1]. In response to these challenges, genetically modified organism (GMO) technology has emerged as an important adaptation strategy. Since its expansion in 1996, GMO cultivation has facilitated the development of climate-resilient crop varieties, and in 2023, global commercial cultivation of GMO crops spanned 206.3 million hectares across 27 countries [2].

Despite the potential benefits of GMOs in addressing climate change, regulatory policies governing their adoption remain fragmented and politically sensitive. The United States has generally taken a more permissive approach, whereas many European countries have implemented strict regulatory barriers. As early adopters and regulatory leaders, both regions have shaped global policy norms for GMO cultivation and trade. However, these divergent approaches create challenges for other countries, which must navigate how to engage in trade with partners that have conflicting GMO regulations [3]. The lack of regulatory alignment not only undermines efforts toward international harmonization but also contributes to instability in global supply chains [4–7].

This paper investigates a key question: how and why do climate shocks influence decisions to approve GMOs, particularly in the context of varying national regulations, technological capabilities, and strategic priorities. Previous research has explored how climate change affects food security [8], the drivers of technological innovation [9–11], and the effects of climate-related risks on global trade [12]. Yet, much less is known about how climate shocks interact with national policies governing GMO approvals. For example, prior study finds rising environmental concerns can increase firms’ incentives to develop cleaner technologies [10]. Similarly, innovation activity intensifies in response to temperature shocks [13]. However, the institutional and economic factors shaping the regulatory approval of GMOs remain largely unexplored. The global community faces a dual challenge: intensifying climate shocks and persistent public debate over GMO safety and acceptance—both of which have implications for food security [14]. This study contributes to closing this gap by examining how climate shocks influence GMO approval patterns through the lens of comparative advantage.

To explore these issues, this paper develops a theoretical model that connects how countries produce and consume agricultural products, with a focus on GMO adoption. On the consumption side, we use a constant elasticity of substitution (CES) utility function to represent how countries consume products with varying levels of GMO content. On the production side, the model considers how differences in resources and exposure to climate shocks affect producers’ decisions to use GMO technology. The key insight from the model is that climate shocks can alter a country’s technological strengths, known as comparative advantage, which in turn shapes its willingness or ability to approve new GMO products. In particular, the model predicts that countries with weaker technological capacity in GMOs may be less inclined to approve them following climate shocks, while countries with stronger capacity are more likely to expand GMO use in response to these challenges.

In the empirical analysis, we test whether countries alter their GMO approval processes in response to climate shocks, employing local projection methods. This study collects GMO approval data at both the country and commodity levels from the International Service for the Acquisition of Agri-biotech Applications (ISAAA) and compile climate shock data from the International Disaster Database (EM-DAT) for the period 2000–2018. To account for differences in technological and economic capacity, we include measures such as GDP per capita and a revealed comparative advantage (RCA) index, which reflect a country’s capacity to adopt GMO technologies. The results show that countries with a weaker comparative advantage in GMOs face more persistent declines in approvals after climate shocks. For example, four years after a shock, GMO approvals drop by an average of 0.037 events in these countries, while no significant change occurs in countries with stronger comparative advantages in GMOs.

This paper contributes to the literature on GMO policy and climate change by offering a novel framework for understanding how climate shocks influence GMO approval processes through their effects on comparative advantage. The findings provide important insights for policymakers, emphasizing that restrictive GMO regulations may amplify the negative consequences of climate shocks—worsening food insecurity and hindering the adoption of critical agricultural innovations needed for climate resilience.

The remainder of the paper is organized as follows. Section 2 develops the conceptual framework and theoretical model. Section 3 and 4 describe the data and methodology. Section 5 presents the empirical findings on the effects of climate shocks on GMO approvals. Section 6 explores empirically the mechanism by examining the role of comparative advantage in GMO technology, and Section 7 is robustness check. Finally, Section 8 is the conclusion.

## 2. Model

### 2.1. Consumption

Consider a two-country two-sector model. Following prior works [15–17], this model assumes a constant elasticity of substitution (CES) demand function with an elasticity of substitution τ∈(0,+∞) over GMO and non-GMO sectors. Consumers in country j maximize


$$U_{j}=C_{j}^{\text{0}}+{\left(C_{j}^{s}\right)}^{\tau }{\left(C_{j}^{-s}\right)}^{\text{1}-\tau }$$ (1)

where $C_{j}^{s}$ aggregates GMO commodity demand and $C_{j}^{-s}$ denotes non-GMO commodity demand. The model assumes consumption relating to GMO (l=s) and non-GMO (l=−s) sectors is substitutable. The consumption quantity of sector l (i.e., l∈s,−s) is subject to a budget constraint


$$\begin{array}{c} s.t.\sum {\mathrm{\backslash nolimits}}_{l}p_{j}^{l}C_{j}^{l}=I_{j} \end{array}$$ (2)

where $I_{j}$ is the nominal consumption income of country j. This model assumes $p_{j}^{l}$ denotes the factory-gate price of sector l in country j’s market. The utility maximization problem solves that


$$C_{j}^{s}=\tau \frac{I_{j}}{p_{j}^{s}};C_{j}^{-s}=(\text{1}-\tau )\frac{I_{j}}{p_{j}^{-s}}$$ (3)

### 2.2. Production and climate shocks

On the supply side, this model assumes producers are price setter under monopolistic competition. A producer maximizes its profit by allocating capital to agricultural technology adoption (e.g., GM innovation and research) and hiring labor. A producer’s profit in sector l in country i is shown as


$${\text{max}}_{K_{i}^{l},L_{i}^{l}}\pi_{i}^{l}=p_{i}^{l}n_{i}^{l}A_{i}^{l}{K_{i}^{l}}^{\alpha }{L_{i}^{l}}^{\text{1}-\alpha }-r_{i}K_{i}^{l}-w_{i}L_{i}^{l}$$ (4)

where α is the elasticity of production with respect to capital, $A_{i}^{l}\;$denotes total factor productivity (TFP) in sector l, and $n_{i}^{l}(\omega_{i}$) denotes the quantitative exercise of productivity affected by climate shocks $\omega_{i}$ ($\frac{\partial n_{i}^{l}(\omega_{i}}{\partial \omega_{i}}<\text{0}$). $A_{i}^{l}n_{i}^{l}\;$denotes the productivity affected by climate shocks in sector l. $r_{i}$ and $w_{i}$ are the unit cost of technology innovation and hiring labor, respectively. This model assumes that country i’s endowment of capital can move between GMO and non-GMO sectors such that the market clearing condition for labor is $\overset{―}{K_{i}^{s}}+\overset{―}{K_{i}^{-s}}=\text{1}$ and relative capital between GMO and non-GMO sectors is $\theta_{i}=K_{i}^{s}/K_{i}^{-s}$; accordingly, $K_{i}^{s}=\frac{\theta_{i}}{\text{1}+\theta_{i}}$. The model also assumes that country i’s labor in both sectors is $\overset{―}{L_{i}^{s}}=\overset{―}{L_{i}^{-s}}=\text{1}$. The reason this paper differentiates GMO and non-GMO sectors in terms of capital allocation is although the production processes of using GMO or non-GMO are very similar, the research and innovation intensity of GM technology (e.g., transgenic plant breeding) is distinct in both sectors, for example, GMO sector is more subject to investments in research, technology, and infrastructure [18]. Thus, this paper emphasizes that Equation (4) replies on the technology adoption process where a country with different $\theta_{i}$ earns distinct profits in conventional and GMO sectors. The relative prices of two sectors is $\frac{p_{i}^{s}}{p_{i}^{-s}}=\frac{n_{i}^{-s}A_{i}^{-s}\alpha {\left(\frac{L_{i}^{-s}}{K_{i}^{-s}}\right)}^{\text{1}-\alpha }}{n_{i}^{s}A_{i}^{s}\alpha {\left(\frac{L_{i}^{s}}{K_{i}^{s}}\right)}^{\text{1}-\alpha }}=\frac{n_{i}^{-s}}{n_{i}^{s}}\frac{A_{i}^{-s}}{A_{i}^{s}}{\left(\theta_{i}\;\right)}^{\text{1}-\alpha }$.

In terms of prices, following prior work [15], the model assumes countries differ in their relative productivities across both sectors in both countries, and normalize


$${\left(q^{s}\right)}^{\eta }{\left(q^{-s}\right)}^{\text{1}-\eta }=\text{1}$$ (5)

The prices of GMO and non-GMO consumption basket thus can be numeraire ${\left(p_{i}^{s}\right)}^{\eta }{\left(p_{i}^{-s}\right)}^{\text{1}-\eta }=\text{1}$, and prices are equal to


$$\left\{ \begin{array}{c} p_{i}^{s}={\left(\frac{n_{i}^{-s}}{n_{i}^{s}}\frac{A_{i}^{-s}}{A_{i}^{s}}\right)}^{\text{1}-\eta }{\left(\theta_{i}\;\right)}^{\left(\text{1}-\alpha )(\text{1}-\eta \right)}\\ p_{i}^{-s}={\left(\frac{n_{i}^{-s}}{n_{i}^{s}}\frac{A_{i}^{-s}}{A_{i}^{s}}\right)}^{-\eta }{\left(\theta_{i}\;\right)}^{-(\text{1}-\alpha )\eta } \end{array}\right.\;$$ (6)

The first order condition of $\pi_{i}^{l}$ with respect to $K_{i}^{l}$ yields that $\frac{r_{i}}{p_{i}^{l}}=n_{i}^{l}A_{i}^{l}\alpha {\left(\frac{L_{i}^{l}}{K_{i}^{l}}\right)}^{\text{1}-\alpha }$, where $p_{i}^{l}$ is the aggregate price of sector l in country i. Combining Equation (6), the return to capital is obtained as


$$r_{i}=n_{i}^{s}A_{i}^{s}\alpha {\left(\frac{\text{1}+\theta_{i}}{\theta_{i}}\;\right)}^{\text{1}-\alpha }{\left(\frac{n_{i}^{-s}}{n_{i}^{s}}\frac{A_{i}^{-s}}{A_{i}^{s}}\right)}^{\text{1}-\eta }{\left(\theta_{i}\;\right)}^{\left(\text{1}-\alpha )(\text{1}-\eta \right)}$$ (7)

Similarly, the first order condition of $\pi_{i}^{l}$ with respect to $L_{i}^{l}$ yields $\frac{w_{i}}{p_{i}^{l}}=n_{i}^{l}A_{i}^{l}(\text{1}-\alpha ){\left(\frac{K_{i}^{l}}{L_{i}^{l}}\right)}^{\alpha }$. The relative prices of two sectors is $\frac{p_{i}^{s}}{p_{i}^{-s}}=\frac{n_{i}^{-s}A_{i}^{-s}{\left(\frac{K_{i}^{-s}}{L_{i}^{-s}}\right)}^{\alpha }}{n_{i}^{s}A_{i}^{s}{\left(\frac{K_{i}^{s}}{L_{i}^{s}}\right)}^{\alpha }}=\frac{n_{i}^{-s}A_{i}^{-s}}{n_{i}^{s}A_{i}^{s}}{\left(\frac{\text{1}}{\theta_{i}}\right)}^{\alpha }$. Labor wage is


$$w_{i}=n_{i}^{s}A_{i}^{s}(\text{1}-\alpha ){\left(\frac{\theta_{i}}{\text{1}+\theta_{i}}\right)}^{\alpha }{\left(\frac{n_{i}^{-s}A_{i}^{-s}}{n_{i}^{s}A_{i}^{s}}\right)}^{\text{1}-\eta }{\left(\frac{\text{1}}{\theta_{i}}\right)}^{\alpha (\text{1}-\eta )}$$ (8)

### 2.3. Market clearance

For market clearance, the total expenditure of sector l in both countries is equal to the total income derived from labor wages and capital returns in both countries. Given the Cobb-Douglas specification of the consumption bundle in Equation (3), total expenditure in the GMO and non-GMO sectors can be expressed as: $p_{j}^{s}C_{j}^{s}=\tau [\sum_{i}\left(r_{i}K_{i}^{s}+w_{i}\right)]$ and $p_{j}^{-s}C_{j}^{-s}=(\text{1}-\tau )[\sum_{i}\left(r_{i}K_{i}^{s}+w_{i}\right)]$. Market clearance also implies aggregate consumption of sector l equalizes aggregate production [19], that is,


$$\begin{array}{c} \sum_{i}\left[p_{i}^{S}A_{i}^{S}{K_{i}^{S}}^{\alpha }{L_{i}^{S}}^{\text{1}-\alpha }\right]=\tau [\sum {\mathrm{\backslash nolimits}}_{i}\left(r_{i}K_{i}^{s}+w_{i}\right)] \end{array}$$ (9)

This model defines country i’s technological or Ricardian comparative advantage in non-GMO commodities as $\gamma_{i}=\frac{n_{i}^{-s}A_{i}^{-s}/n_{i}^{s}A_{i}^{s}}{n_{j}^{-s}A_{j}^{-s}/{n_{j}^{s}A}_{j}^{s}}$.

To close the model, we substitute Equations (6–8) into Equation (9), allowing us to derive an explicit condition for market clearance in terms of productivity, factor endowments, and trade expenditure shares in the two-country setup. Therefore, Equation (9) can be rewritten as


$$\frac{n_{i}^{s}A_{i}^{s}}{n_{j}^{s}A_{j}^{s}}{\left(\gamma_{i}\right)}^{\text{1}-\eta }{\left(\theta_{i}\;\right)}^{\left(\text{1}-\alpha )(\text{1}-\eta \right)}{\left(\frac{\theta_{i}}{\text{1}+\theta_{i}}\right)}^{\alpha }+{\left(\theta_{j}\;\right)}^{\left(\text{1}-\alpha )(\text{1}-\eta \right)}{\left(\frac{\theta_{j}}{\text{1}+\theta_{j}}\right)}^{\alpha }=$$

$$\begin{array}{cc} & \tau_{i}\left[\alpha {\left(\frac{\text{1}+\theta_{i}}{\theta_{i}}\;\right)}^{-\alpha }\frac{n_{i}^{s}A_{i}^{s}}{n_{j}^{s}A_{j}^{s}}{\left(\gamma_{i}\right)}^{\text{1}-\eta }{\left(\theta_{i}\;\right)}^{\left(\text{1}-\alpha )(\text{1}-\eta \right)}+(\text{1}-\alpha ){\left(\frac{\theta_{i}}{\text{1}+\theta_{i}}\right)}^{\alpha }\frac{n_{i}^{s}A_{i}^{s}}{n_{j}^{s}A_{j}^{s}}{\left(\gamma_{i}\right)}^{\text{1}-\eta }{\left(\frac{\text{1}}{\theta_{i}}\right)}^{\alpha (\text{1}-\eta )}\right]\\ & +\tau_{j}\left[\alpha {\left(\frac{\text{1}+\theta_{j}}{\theta_{j}}\;\right)}^{-\alpha }{\left(\theta_{j}\;\right)}^{\left(\text{1}-\alpha )(\text{1}-\eta \right)}+(\text{1}-\alpha ){\left(\frac{\theta_{j}}{\text{1}+\theta_{j}}\right)}^{\alpha }{\left(\frac{\text{1}}{\theta_{j}}\right)}^{\alpha (\text{1}-\eta )}\right] \end{array}$$ (10)

**Fig 1:**
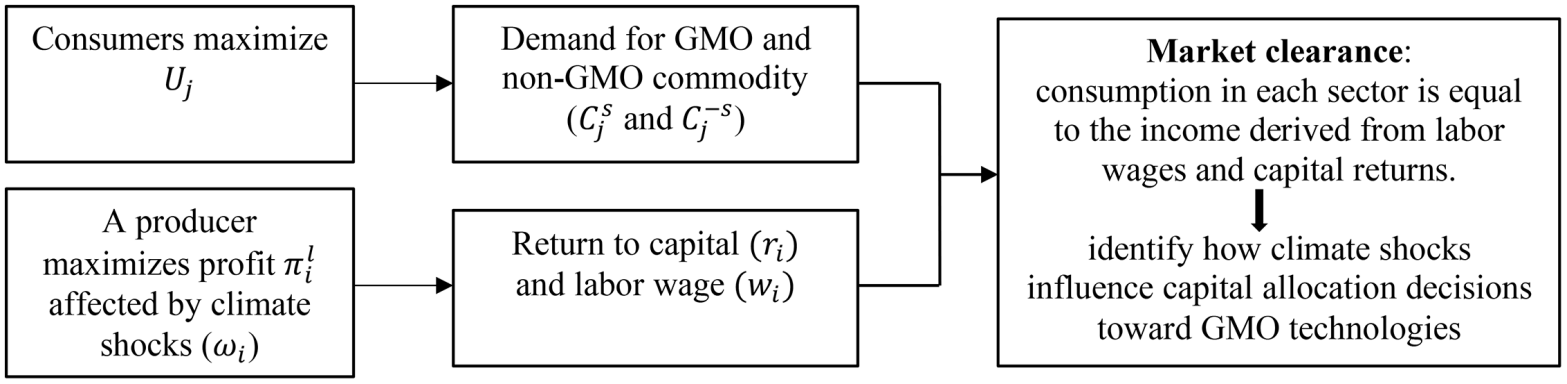
A diagrammatic representation of the structural model.

In simple terms, our model operates in three steps. First, utility maximization on the consumer side yields optimal demand for GMO and non-GMO commodities. Second, on the production side, the producer’s profit function—subject to climate shocks—determines the optimal return to capital and labor wages. Finally, under market clearance, where total consumption equals the total income from capital returns and labor wages, the equilibrium can be expressed using countries’ comparative advantages in GMO and non-GMO commodities. This structure allows us to identify how climate shocks influence producers’ capital allocation decisions toward GMO technologies through changes in comparative advantage. Fig 1 presents a diagrammatic representation of this structural model.

### 2.4. Theoretical mechanism and proposition

This section examines how climate shocks ($\omega_{i}$) affect country i’s comparative advantage in GMO ($\frac{\text{1}}{\gamma_{i}}$) and how this shift influences capital allocation to the GMO sector.

**Proposition 1** Holding other countries’ productivity constant, if climate shocks reduce country’s comparative advantage in GMO goods, then country has less incentive to allocate capital to the GMO sector.

**Proof of Proposition 1** Taking the derivative of $\frac{\text{1}}{\gamma_{i}}$ with respect to the climate shock $\omega_{i}$, we get:


$$\frac{\partial \frac{\text{1}}{\gamma_{i}\left(\omega_{i}\right)}}{\partial \omega_{i}}=\frac{\partial \left(\frac{\frac{n_{i}^{s}\left(\omega_{i}\right)A_{i}^{s}}{n_{i}^{-s}\left(\omega_{i}\right)A_{i}^{-s}}}{\frac{{n_{j}^{s}A}_{j}^{s}}{n_{j}^{-s}A_{j}^{-s}}}\right)}{\partial \omega_{i}}=\frac{\text{1}}{\frac{{n_{j}^{s}A}_{j}^{s}}{n_{j}^{-s}A_{j}^{-s}}}\frac{\partial \left(n_{i}^{s}\left(\omega_{i}\right)A_{i}^{s}\right)}{\partial \omega_{i}}\frac{\partial \left({\left(n_{i}^{-s}\left(\omega_{i}\right)A_{i}^{-s}\right)}^{-\text{1}}\right)}{\partial \omega_{i}}=-{\text{H}}_{i}\frac{\partial \left(n_{i}^{-s}\left(\omega_{i}\right)A_{i}^{-s}\right)}{\partial \omega_{i}}\frac{\partial \left(n_{i}^{s}\left(\omega_{i}\right)A_{i}^{s}\right)}{\partial \omega_{i}}<\text{0}$$ (11)

where ${\text{H}}_{i}={\left(n_{i}^{-s}\left(\omega_{i}\right)A_{i}^{-s}\right)}^{-\text{2}}\frac{\text{1}}{\frac{{n_{j}^{s}A}_{j}^{s}}{n_{j}^{-s}A_{j}^{-s}}}$. This derivative in Equation (11) is negative, indicating that climate shocks reduce country’s comparative advantage in GMO goods.

**Fig 2:**
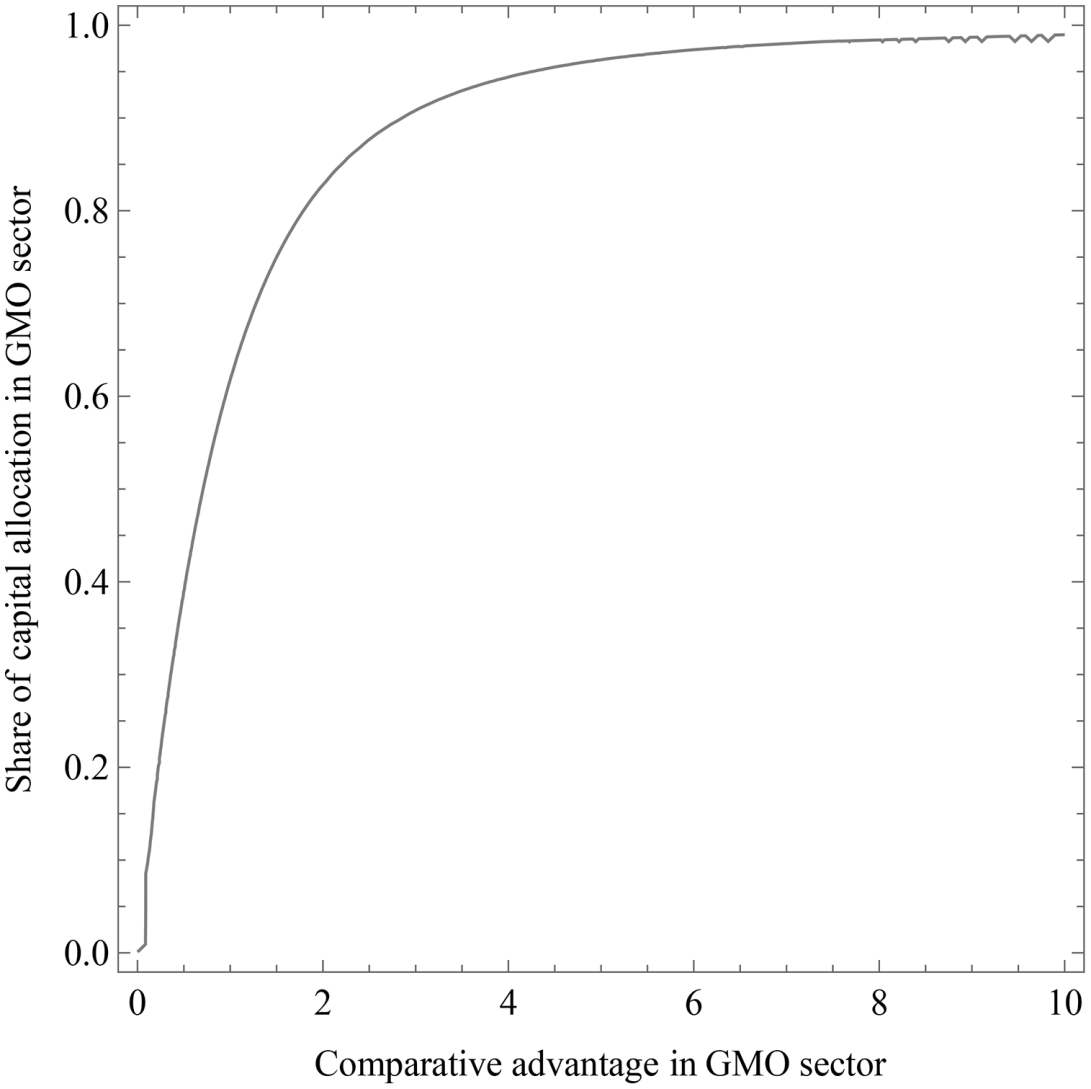
The impact of comparative advantage in the GMO sector on GMO adoption.

**Fig 3:**
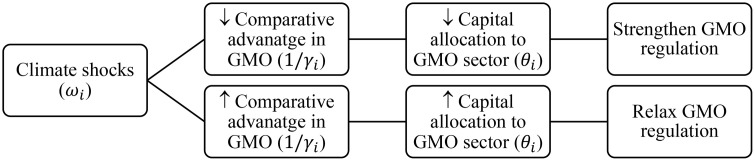
Mechanism linking climate shocks to GMO regulation.

We then explore how comparative advantage in GMOs, $\frac{\text{1}}{\gamma_{i}}$, influences the capital allocation decision to GMO-intensive production, $\theta_{i}$. Since a closed-form solution for capital allocation $\theta_{i}$ is not analytically tractable, we employ numerical simulations in Mathematica by assuming other model parameters constant (i.e., α,η, $\tau_{i},\tau_{j},\;\theta_{j}$). Parameter values are set as follows: α= 0.5, $\tau_{i}=\tau_{j}=\text{0}.\text{4}$, η= 0.5$\theta_{j}=\text{10}$. Fig 2 plots $\theta_{i}$ against $\frac{\text{1}}{\gamma_{i}}$, showing that countries with lower comparative advantage in GMO production allocate less capital to the GMO sector ($\frac{\partial \theta_{i}}{\partial \frac{\text{1}}{\gamma_{i}}}<\text{0}$). This suggests that countries lacking a comparative advantage in GMOs may hesitate to invest in GMO technologies due to high adaptation costs, institutional constraints, or limited capacity. These findings are consistent with Conte et al. (2021), who argue that countries tend to reallocate resources toward sectors where they retain relative competitiveness following external shocks. Fig 3 also depicts the mechanism linking climate Shocks to GMO regulatary regimes.

### 2.5. From theory to data

#### Core theoretical hypothesis.

Climate shocks reduce comparative advantage in GMO production and decrease capital allocated to GMO technologies.

#### Empirical hypothesis.

Climate shocks lead to less GMO approvals in countries with a comparative disadvantage in GMOs.

Our theoretical results provide a transparent framework for linking theory to data and guiding the empirical analysis, which proceeds in three parts. First, climate shocks ($\omega_{i}$)—treated as exogenous in the theoretical model—are empirically measured using standardized event counts from the EM-DAT disaster database. Second, GMO approvals ($\theta_{i}$) in the theory are proxied in the empirical analysis by the number of GMO events approved in each country-year. Third, comparative advantage in GMOs ($\frac{1}{\gamma_{i}}$) in the theory is operationalized in two empirical ways. The first proxy is GDP per capita, which reflects a country’s capacity to invest in and adopt GMO innovations. The second is a Revealed Comparative Advantage (RCA) index, which measures whether a country’s export specialization in GMO products exceeds its overall export performance [3]. This index closely aligns with the theoretical concept of specialization and capability in GMO development ($\frac{1}{\gamma_{i}}$i).

## 3. Data

Climatic shock data is collected from the International Disaster Database (EM-DAT) for the period 2000–2018. For a shock to be entered into the database at least one of the following criteria must be fulfilled: ten or more people reported killed, one hundred or more people reported affected, declaration of a state of emergency, or call for international assistance [19]. The dataset has been widely used in previous studies on climatic shocks [20–23]. The shock types include geophysical, meteorological, hydrological, climatological, biological, and extra-terrestrial. In this study, climate shocks include droughts, heat waves, cold waves, severe winter conditions, floods, and storms. Cold wave includes a period of abnormally cold weather. For example, according to EM-DAT, heat wave refers a period of abnormally hot and/or unusually humid weather that lasts for two or more days. Severe winter conditions are damage caused by snow and ice. Winter damage refers to damage to buildings, infrastructure, traffic (esp. navigation) inflicted by snow and ice in the form of snow pressure, freezing rain, frozen waterways etc. Drought refers an extended period of unusually low precipitation that produces a shortage of water for people, animals, and plants.

GMO approval data is collected from the ISAAA, containing all GMO events at the commodity and country levels each year. Since GMO and non-GMO crops cannot be distinguished and not available in the dataset, this paper considers industries that include the most commonly GMO crops—namely, cotton, maize, soybeans, and rapeseed—which continually develop advanced GMO traits. Thus, the GMO use in the selected crops reflects countries’ adoption behavior of GMOs.

Based on prior work [24,25], the revealed comparative advantage (RCA) index is calculated by collecting data from the United Nations (UN) Comtrade, which consists of the value of bilateral exports at the commodity and country levels in current (rather than constant) dollars. The products are defined according to the SITC Revision 3 aggregates: soybeans (2222), maize (044), cotton seeds (2223), and rape or colza seeds (22261).

The control variables include agricultural share, arable land per capita, GMO area, infrastructure conditions, an international agreement indicator, infrastructure conditions, and a developing country dummy. Many countries adopt alternative adaptation strategies such as fertilizer use and infrastructure investment. In the theoretical model, these are treated as fixed, non-technology-specific costs that do not affect the marginal returns to capital and labor across GMO and non-GMO sectors, and thus do not affect the optimal GMO allocation decision derived from the first-order conditions of Equation (4). Empirically, we control for adaptive capacity using the ND-GAIN infrastructure index. Fertilizer use is excluded due to limited disaggregated data and potential endogeneity, as it may correlate with unobserved factors such as crop composition or technological capacity.

Data on agricultural share and arable land per capita are collected from World Bank Open Data. Annual biotech acreage in a million hectares is obtained from existing work [26]. The infrastructure score is generated by the Notre Dame Global Adaptation Index (ND-GAIN), which measures the vulnerability of the infrastructure sector. The infrastructure score, ranging from 0 to 1, combines the indicators including projected change in hydropower generation capacity, projected change of sea level rise impacts, dependence on imported energy, population living under 5m above sea level, electricity access, and disaster preparedness. A higher indicator means the infrastructure is more impacted by certain shock. The international agreement indicator, collected from prior work [27], measures whether a country subscribes to the two GMO-specific international agreements, i.e., Codex Alimentarius and the Cartagena Protocol on Biosafety. The UPOV indicator measured by prior work [28] measures country adherence to revisions of the International Convention for the Protection of New Varieties of Plants (UPOV). Tables 1 and 2 report the descriptive statistics for all estimated variables.

**Table 1:** Descriptive statistics for number of climate shocks and control variables.

	Obs	Mean	St. Dev.	Min	Max
*Number of climate shocks*					
All	5168	3.04	4.59	0	32
Cold wave	5168	0.12	0.37	0	3
Severe winter	5168	0.03	0.17	0	2
Flood	5168	1.43	2.27	0	20
Heatwave	5168	0.09	0.29	0	3
Drought	5168	0.11	0.34	0	3
Storm	5168	1.04	2.49	0	19
*Controls*					
Agricultural share	5164	7.33	8.74	0.03	57.14
UPOV dummy	5168	0.69	0.46	0	1
Infrastructure	4864	0.35	0.1	0.14	0.64
Developing country dummy	5168	0.53	0.5	0	1
Arable Land	5120	0.27	0.26	0.00	1.42
Log(GDP)	5168	9.11	1.42	4.72	11.73

**Table 2:** Descriptive statistics for number of GMO approvals (*Obs = 5168*).

	Mean	St. Dev.	Min	Max
All approvals	0.77	1.75	0	18
*Trait*				
Insect Resistant	0.45	1.46	0	18
Herbicide Tolerance	0.66	1.58	0	15
Drought Tolerance	0.01	0.14	0	4
Yield Improvement	0.00	0.01	0	1

Note. The most widely used GMO traits thus far involve herbicide tolerance and insect resistance (Qaim 2020).

## 4. Methodology

Following existing studies [29,30], this paper employs the local projection method to estimate the cumulative impacts of climate shocks on GMO approval events, rather than focusing on marginal effects. The advantage of using local projections lies in their flexibility: they estimate the dynamic response of an outcome at different time horizons without requiring strong assumptions about the underlying data-generating process [31]. This approach is particularly suitable in our context, where previously approved GMO events may influence future approvals due to the long-term utility of these technologies. By estimating impulse response functions directly, the method accounts for the dynamic nature of policy adoption while avoiding the complexity of specifying and estimating a full vector autoregressive (VAR) model.

Following prior work [31], the empirical model regresses the level of the outcome (number of GMO appovals) at horizon h (i.e., t+h) on its lagged values and current covariates, i.e., climate shocks, enabling us to trace out the effect of a shock over time. The impulse response is estimated as the following model:


$$\theta_{ij,t+h}=\beta_{\text{1}}^{h}\omega_{i,t}+\beta_{\text{3}}^{h}(\theta_{ij,t-\text{1}}\;)+\beta_{\text{4}}^{h}\sigma_{i}+\delta_{j}^{h}+\varepsilon_{i,t}^{h}$$ (12)

where $\omega_{i,t}$ denotes the number of shocks in country i at year t, which is the main variable of interest in this paper. The dependent variable is $\theta_{ij,t+h}$ which denotes the number of GMO approvals in levels at horizons t+h for product j. Horizon h is the estimation time period, ranging from horizon 0 to horizon 7, which therefore captures the contemporaneous and cumulative effects up to 7 years after the shock. The coefficient $\beta_{\text{1}}^{h}$ captures the impact of one additional climate shock on GMO approvals *h* years later. The lagged dependent variable $\theta_{ij,t-\text{1}}$ controls for prior approval trends.

To address potential endogeneity, the analysis adopts several strategies. First, climate shocks—such as droughts, floods, or extreme cold—are treated as plausibly exogenous, as defined by EM-DAT’s physical criteria, and are unlikely to be driven by GMO policy. Second, the local projection framework helps mitigate simultaneity concerns by estimating delayed effects of shocks over time, which aligns with the regulatory nature of GMO approval processes.

Third, the model includes a set of country-year control variables $\sigma_{i,t}$ to address omitted variable bias. These controls are selected for their presumed exogeneity and relevance to GMO regulatory capacity. Specifically, the model includes: (1) an indicator for membership in the International Union for the Protection of New Varieties of Plants (UPOV), which reflects alignment with global biosafety and intellectual property frameworks such as the Codex Alimentarius and the Cartagena Protocol on Biosafety; (2) the agricultural share of GDP; (3) arable land per capita; and (4) an index of infrastructure quality. These variables capture differences in regulatory institutions, agricultural dependence, and a country’s capacity to adopt and implement GMO technologies. Prior research indicates that countries with weaker infrastructure or a higher reliance on agriculture may respond differently to climate shocks in the context of GMO adoption [32–35]. Other potentially relevant variables, such as fertilizer use, are excluded due to endogeneity concerns. Fertilizer application may reflect unobserved factors—such as crop mix, farming practices, or technological capacity—that also affect GMO approval decisions. Including such variables could introduce multicollinearity with existing variables or bias the estimated relationship between climate shocks and regulatory responses.

Forth, the country fixed effect $\sigma_{i}$ absorb unobserved, time-invariant heterogeneity such as climate, geography, dietary preferences, or baseline regulatory stances. Commodity fixed effects $\delta_{j}^{h}$ account for persistent differences across products—for example, cotton may require fewer GMO traits than maize. Standard errors are clustered at the country-product level to allow for serial correlation and within-cluster heteroskedasticity.

## 5. Results

To assess the long-term impact of climate shocks on GMO approvals, Table 3 presents cumulative effects from the first year up to seven years after a shock. These results incorporate lag structures and control variables, capturing how an additional climate shock influences the number of GMO approvals over time. In the first panel, which includes country and commodity fixed effects but omits control variables, we find that climate shocks significantly reduce GMO approvals four years after the event. Effects in later years are statistically insignificant. In the second panel, which adds key control variables, the effect remains: a single shock is associated with a 0.031-unit decline in approvals in year four. This timing aligns with average GMO approval timelines, typically ranging from 1.75 to 5.9 years [36]. Among the controls, a higher agricultural share of GDP is negatively associated with approvals in years six and seven, suggesting that countries with smaller agricultural sectors may be more inclined to adopt GMOs to address productivity concerns. UPOV membership is positively correlated with approvals, reinforcing the role of international institutional alignment in facilitating regulatory adoption.

**Table 3:** Local projections of the cumulative effects on GMO approvals relative to total climate shocks (Up to 7 Years Post-Shock).

	Horizon 1	Horizon 2	Horizon 3	Horizon 4	Horizon 5	Horizon 6	Horizon 7
Total Shock	−0.009	−0.019	−0.001	−0.035*	0.008	−0.015	0.000
	(0.012)	(0.013)	(0.014)	(0.014)	(0.017)	(0.014)	(0.017)
Lag_Approval	0.138***	0.351***	0.143***	0.020	0.234***	0.062*	−0.041
	(0.021)	(0.031)	(0.027)	(0.018)	(0.038)	(0.031)	(0.032)
Observations	4624	4352	4080	3808	3536	3264	2992
R-squared	0.232	0.33	0.265	0.262	0.295	0.285	0.288
Total Shock	−0.004	−0.015	0.002	−0.031*	0.011	−0.014	0.003
	(0.012)	(0.012)	(0.015)	(0.014)	(0.016)	(0.015)	(0.018)
Lag_Approval	0.129***	0.334***	0.134***	0.008	0.220***	0.052	−0.049
	(0.022)	(0.031)	(0.028)	(0.019)	(0.038)	(0.031)	(0.034)
Agri Share	−0.031	−0.034	−0.05	−0.054	−0.043	−0.037*	−0.050*
	(0.018)	(0.018)	(0.026)	(0.028)	(0.022)	(0.018)	(0.022)
Arable Land	0.922	0.169	0.386	0.319	1.076	(0.057)	0.490
	(0.995)	(0.587)	(0.675)	(0.766)	(1.008)	(1.120)	(0.923)
UPOV	0.526**	0.574**	0.605***	0.640***	0.346*	0.217	0.381***
	(0.175)	(0.181)	(0.162)	(0.158)	(0.136)	(0.113)	(0.109)
Infrastructure	2.366	3.160	4.990	4.043	4.398*	4.600	4.193
	(2.040)	(1.878)	(2.644)	(3.104)	(1.983)	(2.322)	(2.216)
Observations	4308	4052	3796	3540	3284	3028	2772
R-squared	0.234	0.329	0.27	0.267	0.295	0.285	0.29

Note. The dependent variable is the number of GMO events approved, so coefficients are interpreted in unit terms. Horizons refer to the response years. Standard errors are in parentheses. Statistical significance is denoted by *** (1%), ** (5%), and * (10%) levels. Country fixed effect and commodity fixed effect are included.

Table 4 breaks down effects by the type of climate shock—cold waves, severe winters, heatwaves, droughts, floods, and storms—while controlling for country, commodity, and other variables. Cold waves exhibit the most sustained impact: one additional cold wave leads to a 0.290-unit increase in GMO approvals by year seven. Conversely, droughts exhibit a delayed but negative effect, significantly reducing approvals in years two and six. Severe winter events (including snow, ice, frost, and freeze) are linked to a cumulative 0.264-unit reduction in approvals over the seven-year window.

**Table 4:** Local projections of the cumulative effects on GMO approvals relative to separate climate shocks (Up to 7 Years Post-Shock).

	Horizon 1	Horizon 2	Horizon 3	Horizon 4	Horizon 5	Horizon 6	Horizon 7
Cold wave	0.171*	−0.269***	0.304**	0.031	0.188*	−0.217**	0.290*
	(0.075)	(0.073)	(0.098)	(0.079)	(0.076)	(0.072)	(0.112)
Observation	4308	4052	3796	3540	3284	3028	2772
R-squared	0.235	0.331	0.273	0.266	0.296	0.286	0.292
Drought	0.025	−0.170*	−0.029	−0.014	0.037	−0.266***	−0.074
	(0.088)	(0.067)	(0.076)	(0.070)	(0.069)	(0.060)	(0.167)
Observation	4308	4052	3796	3540	3284	3028	2772
R-squared	0.234	0.329	0.27	0.266	0.295	0.286	0.29
Flood	−0.006	−0.006	−0.026	−0.020	0.003	0.000	0.009
	(0.017)	(0.015)	(0.022)	(0.018)	(0.019)	(0.020)	(0.022)
Observation	4308	4052	3796	3540	3284	3028	2772
R-squared	0.234	0.328	0.271	0.266	0.295	0.285	0.29
Heat wave	−0.113	0.002	−0.161	0.095	−0.024	0.142	0.023
	(0.115)	(0.082)	(0.123)	(0.117)	(0.083)	(0.129)	(0.138)
Observation	4308	4052	3796	3540	3284	3028	2772
R-squared	0.235	0.328	0.271	0.266	0.295	0.285	0.29
Severe winter condition	−0.389***	0.280**	−0.147**	−0.110	0.334***	0.175	−0.264***
	(0.107)	(0.092)	(0.047)	(0.077)	(0.096)	(0.155)	(0.061)
Observation	4308	4052	3796	3540	3284	3028	2772
R-squared	0.236	0.329	0.271	0.266	0.296	0.285	0.29
Storm	−0.003	0.002	0.017	−0.092**	−0.027	−0.016	−0.032
	(0.025)	(0.028)	(0.021)	(0.031)	(0.036)	(0.034)	(0.042)
Observation	4308	4052	3796	3540	3284	3028	2772
R-squared	0.234	0.328	0.27	0.269	0.295	0.285	0.29

Note. The dependent variable is the number of GMO events approved, so coefficients are interpreted in unit terms. Horizons refer to the response years. Standard errors are in parentheses. Statistical significance is denoted by *** (1%), ** (5%), and * (10%) levels. Country fixed effect and commodity fixed effect are included.

To focus specifically on agriculturally relevant shocks, Table 5 restricts the analysis to climate events occurring in key agricultural areas. These regions are identified by spatially matching disaster locations with the SPAM production map [37]. EM-DAT provides data on disasters at the state level. As the SPAM data is at the commodity level, this paper aligns EM-DAT with SPAM data, specifically identifying countries’ main agricultural regions (i.e., states) by ascertaining each commodity—namely, soybeans, maize, cotton, and rape or colza—exhibits an agricultural yield greater than zero. Consistent with earlier results, cold waves again show the most persistent positive effect on GMO approvals, while droughts notably suppress approvals six years after the shock. These findings emphasize that not all climate shocks lead to adaptive regulatory responses and underscore the importance of spatial and sectoral context in shaping GMO policy outcomes.

**Table 5:** Local projections of the cumulative change in GMO approvals relative to separate climate shocks occurring in main cropping regions (Up to 7 Years Post-Shock).

	Horizon 1	Horizon 2	Horizon 3	Horizon 4	Horizon 5	Horizon 6	Horizon 7
Cold wave	0.268*	−0.220**	0.263*	0.191	0.158	−0.174*	0.215
	(0.111)	(0.080)	(0.114)	(0.122)	(0.096)	(0.070)	(0.148)
Observation	4308	4052	3796	3540	3284	3028	2772
R-squared	0.236	0.33	0.272	0.267	0.296	0.286	0.29
Drought	0.016	−0.218**	−0.050	−0.033	−0.016	−0.262***	−0.095
	(0.110)	(0.081)	(0.083)	(0.085)	(0.088)	(0.073)	(0.196)
Observation	4308	4052	3796	3540	3284	3028	2772
R-squared	0.234	0.329	0.27	0.266	0.295	0.286	0.29
Flood	−0.034	0.012	−0.035	−0.069	0.027	−0.016	−0.012
	(0.024)	(0.022)	(0.027)	(0.045)	(0.035)	(0.033)	(0.040)
Observation	4308	4052	3796	3540	3284	3028	2772
R-squared	0.235	0.328	0.271	0.267	0.295	0.285	0.29
Heat wave	−0.003	0.091	−0.075	0.261	−0.123	0.140	0.256
	(0.109)	(0.120)	(0.102)	(0.172)	(0.107)	(0.109)	(0.215)
Observation	4308	4052	3796	3540	3284	3028	2772
R-squared	0.234	0.329	0.27	0.267	0.295	0.285	0.29
Severe winter condition	−0.018	−0.008	0.003	−0.053	−0.010	−0.057	−0.026
	(0.031)	(0.019)	(0.031)	(0.042)	(0.038)	(0.047)	(0.046)
Observation	4308	4052	3796	3540	3284	3028	2772
R-squared	0.234	0.328	0.27	0.267	0.295	0.286	0.29
Storm	−0.450***	0.483**	−0.324***	−0.039	0.518**	0.003	−0.305***
	(0.108)	(0.152)	(0.067)	(0.095)	(0.157)	(0.226)	(0.081)
Observation	4308	4052	3796	3540	3284	3028	2772
R-squared	0.236	0.33	0.271	0.266	0.297	0.285	0.29

Note. The dependent variable is the number of GMO events approved, so coefficients are interpreted in unit terms. Horizons refer to the response years. Standard errors are in parentheses. Statistical significance is denoted by *** (1%), ** (5%), and * (10%) levels. Country fixed effect and commodity fixed effect are included.

## 6. Mechanism: Comparative advantage of GMOs

In the theoretical model, this paper shows that countries with a strong comparative advantage in developing GMO technologies tend to adopt less restrictive regulatory stances toward GMO crops. In contrast, countries with weaker comparative advantages are generally more restrictive. This section builds on that framework by empirically examining how comparative advantage moderates the relationship between climate shocks and GMO approval decisions.

**Fig 4:**
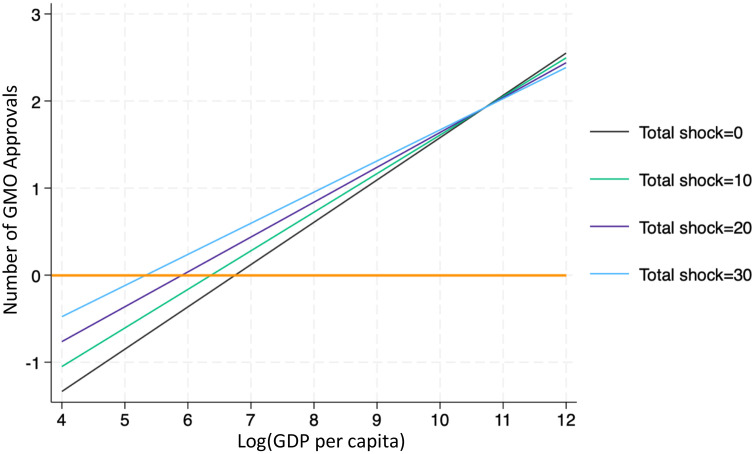
Marginal effects of the logarithm of GDP per capita on GMO approvals at different climate shock frequency levels.

Comparative advantage in GMO technology is measured in two ways. First, the one-year lag of logarithm of a country’s GDP per capita is used as a proxy for its capacity to invest in and adopt GMO innovations. Countries with higher income levels are presumed to have greater technological readiness and institutional support for GMO adoption. To capture this dynamic, the model includes an interaction term between GDP per capita and climate shocks. Table 6 reports the estimated effects, while Fig 4 visualizes the relationship seven years after a shock. The results indicate that GMO approvals increase with GDP per capita. However, at lower income levels, approvals may remain negative following a shock and only turn positive once GDP exceeds a certain threshold. For example, in countries experiencing ten climate shocks in a given year, approvals tend to rise only if GDP per capita is above exp(6.2), or approximately $493. This suggests that limited economic resources and weaker technological capacity may prevent low-income countries from advancing GMO approvals in response to extreme climate events.

**Table 6:** Local projections of the cumulative change in GMO approvals relative to climate shocks (Up to 7 Years Post-Shock) with GMO expertise interaction.

	Horizon 1	Horizon 2	Horizon 3	Horizon 4	Horizon 5	Horizon 6	Horizon 7
Total Shock x Log (GDP per capita)	−0.013	−0.009	−0.009	−0.020***	0.002	0.002	−0.004
	(0.011)	(0.005)	(0.005)	(0.005)	(0.007)	(0.008)	(0.007)
Log (GDP per capita)	0.765***	0.687***	0.766***	0.731***	0.360***	0.380***	0.486***
	(0.111)	(0.095)	(0.098)	(0.100)	(0.080)	(0.077)	(0.086)
Total Shock	0.120	0.074	0.091*	0.144***	−0.002	−0.025	0.046
	(0.086)	(0.052)	(0.039)	(0.037)	(0.066)	(0.059)	(0.058)
Lag_Approval	0.106***	0.313***	0.109***	−0.016	0.206***	0.037	−0.066
	(0.022)	(0.031)	(0.029)	(0.020)	(0.038)	(0.031)	(0.036)
Agri Share	0.052***	0.044**	0.041*	0.028	0.002	0.009	0.006
	(0.013)	(0.013)	(0.016)	(0.020)	(0.018)	(0.016)	(0.020)
Arable Land	0.154	−0.516	−0.382	−0.472	0.826	−0.262	0.339
	(0.972)	(0.511)	(0.584)	(0.869)	(1.126)	(1.200)	(1.160)
UPOV	0.138	0.233	0.229	0.288*	0.178	0.045	0.162
	(0.174)	(0.177)	(0.146)	(0.140)	(0.140)	(0.113)	(0.110)
Infrastructure	6.407***	6.875***	9.044***	7.811**	6.297**	6.486*	6.407*
	(1.578)	(1.700)	(2.212)	(2.402)	(2.082)	(2.448)	(2.529)
Observations	4308	4052	3796	3540	3284	3028	2772
R-squared	0.251	0.342	0.287	0.281	0.299	0.289	0.294

Note. The dependent variable is the number of GMO events approved, so coefficients are interpreted in unit terms. Horizons refer to the response years. Standard errors are in parentheses. Statistical significance is denoted by *** (1%), ** (5%), and * (10%) levels. Country fixed effect and commodity fixed effect are included.

**Fig 5:**
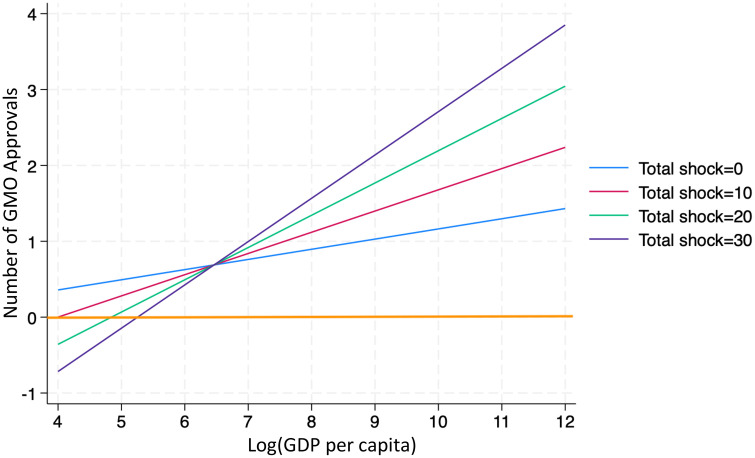
Marginal effects of the logarithm of GDP per capita on GMO approvals that have been approved before.

To further isolate the policy response toward GMO varieties already approved elsewhere, the analysis is repeated using only pre-approved events—those that can typically be fast-tracked due to established safety records. Table 7 and Fig 5 present the results. In countries with GDP per capita above exp(6.5), or about $665, climate shocks are associated with an increase in approvals of pre-cleared GMO events. In contrast, at lower GDP levels, shocks reduce the likelihood of approving even these events. This finding supports the view that capacity constraints—not just regulatory attitudes—shape how countries respond to environmental stress. In low-income settings, even well-established GMO technologies may face additional barriers after a climate shock, while wealthier nations tend to view such shocks as motivation to accelerate innovation.

**Table 7:** Impact of total climate shocks on GMO approvals in first time approvals and have been approved before.

	Horizon 1	Horizon 2	Horizon 3	Horizon 4	Horizon 5	Horizon 6	Horizon 7
	Have been approved before						
Total Shock	−0.056	−0.094	−0.049	−0.063	−0.226*	−0.131	−0.094
	(0.056)	(0.071)	(0.068)	(0.091)	(0.107)	(0.117)	(0.116)
Log (GDP per capita)	0.684***	0.949***	0.866***	0.713***	0.378**	0.253*	0.134
	(0.137)	(0.164)	(0.130)	(0.125)	(0.114)	(0.120)	(0.135)
Total Shock x Log (GDP per capita)	0.007	0.013	0.007	0.007	0.031*	0.011	0.015
	(0.007)	(0.008)	(0.008)	(0.011)	(0.012)	(0.014)	(0.014)
Lag_Approval	0.016	−0.026	0.014	−0.051***	−0.021	−0.034	0.025
	(0.015)	(0.020)	(0.019)	(0.014)	(0.022)	(0.024)	(0.029)
Agri Share	0.035*	0.058**	0.040	0.024	−0.001	0.015	0.006
	(0.016)	(0.020)	(0.020)	(0.022)	(0.021)	(0.025)	(0.024)
Arable Land	−0.022	−1.140	−1.195	−0.968	0.854	−0.330	−0.028
	(1.222)	(0.861)	(0.789)	(1.245)	(1.745)	(1.849)	(1.868)
UPOV	0.304	0.251	0.178	0.308	−0.043	−0.064	0.001
	(0.288)	(0.295)	(0.259)	(0.289)	(0.266)	(0.280)	(0.280)
Infrastructure	7.509***	10.325***	11.550***	9.268**	6.351**	4.366	1.902
	(2.086)	(2.097)	(2.663)	(2.921)	(2.273)	(2.875)	(2.565)
Observations	3832	3604	3376	3148	2920	2692	2464
R-squared	0.166	0.184	0.183	0.178	0.174	0.175	0.173

Note. The dependent variable is the number of GMO events approved, so coefficients are interpreted in unit terms. Horizons refer to the response years. Standard errors are in parentheses. Statistical significance is denoted by *** (1%), ** (5%), and * (10%) levels. Country fixed effect and commodity fixed effect are included.

Second, this paper uses the standard Balassa-based Revealed Comparative Advantage (RCA) index to measure a country’s relative specialization in GMO-related products. RCA is calculated as ${RCA}_{ijt}=\text{log}\frac{E_{ij}}{E_{it}}-\text{log}\frac{E_{-i,j}}{E_{-i,t}}$. $E_{ij}$ denotes the exports of country i in industry j. $E_{it}$ is country i’s total exports. $E_{-i,j}$ indicates all other countries’ exports in industry j, and $E_{-i,t}$ denotes the other countries’ total exports. The RCA compares a country’s share of exports in a given product to its overall export profile. An RCA value greater than zero indicates comparative advantage, while a value less than zero signals disadvantage. The sample is split into two groups—advantaged and disadvantaged—based on the country’s lagged RCA score, ensuring that current shocks do not affect group classification. Using zero as a reference point, the two subsamples are relatively large and comparable in size.

We carefully considered alternative measures of comparative advantage, including the Revealed Competitive Advantage (RC) index [38] and the Revealed Symmetric Comparative Advantage (RSCA) index [38,39] but ultimately chose not to use them. First, the RC index incorporates net trade and import values, capturing overall trade performance rather than export specialization. In our context, however, GMO adoption is more closely tied to a country’s export capacity and engagement with global biotechnology markets. Because the RC index reflects import behavior and trade balances, it risks conflating comparative advantage with trade protection or domestic consumption patterns, which are not the focus of our export-oriented framework. Second, the RSCA index is a normalized transformation of the RCA index intended to improve cross-country comparability by addressing scaling and symmetry. While useful in studies relying on continuous measures, our empirical strategy relies on a binary classification—distinguishing countries with a positive versus negative comparative advantage based on the sign of lagged RCA values. Since RSCA preserves the same directional classification, it does not offer additional analytical value for our purposes.

Controlling for country and commodity fixed effects, Table 8 reports how climate shocks affect GMO approvals in both groups. The results show that the negative effects of shocks are more persistent in countries with a comparative disadvantage. Specifically, four years after a shock, GMO approvals decline by 0.037 events on average in these countries. In contrast, no significant effect is found in countries with a comparative advantage. This suggests that countries lacking specialization in GMO technology may adopt a more protective stance following climate shocks, potentially delaying approvals and contributing to international GMO disputes. Prior studies have similarly noted that restrictive GMO policies can serve as non-tariff barriers in countries lacking specialization in GMOs [33].

**Table 8:** Impact of total climate shocks on GMO approvals in subsamples in terms of comparative advantages.

	Horizon 1	Horizon 2	Horizon 3	Horizon 4	Horizon 5	Horizon 6	Horizon 7
	Comparative advantaged countries						
Total Shock	0.005	−0.005	−0.046	0.000	0.019	−0.01	−0.024
	(0.021)	(0.034)	(0.033)	(0.027)	(0.030)	(0.048)	(0.036)
Lag_Approval	0.086	0.248**	−0.015	−0.101*	0.123	−0.052	−0.046
	(0.086)	(0.078)	(0.057)	(0.041)	(0.120)	(0.068)	(0.079)
Agri Share	−0.109	−0.090	−0.175*	−0.094	−0.007	−0.065	−0.044
	(0.056)	(0.045)	(0.065)	(0.072)	(0.059)	(0.058)	(0.072)
Arable Land	1.091	1.082	0.843	1.334	3.458	1.228	1.891
	(0.951)	(1.180)	(1.275)	(2.091)	(2.588)	(3.024)	(2.002)
UPOV	−0.600	−0.409	−0.379	−0.077	−0.312	−0.488	0.375
	(0.433)	(0.546)	(0.459)	(0.370)	(0.502)	(0.370)	(0.318)
Infrastructure	8.007	7.228	13.141	12.851	2.599	4.798	3.452
	(5.382)	(6.339)	(8.378)	(9.717)	(5.034)	(6.758)	(6.784)
Observations	734	690	646	600	551	504	458
R-squared	0.253	0.305	0.303	0.302	0.290	0.305	0.332
	Comparative disadvantaged countries						
Total Shock	−0.004	−0.017	0.011	−0.037*	0.010	−0.015	0.010
	(0.013)	(0.013)	(0.015)	(0.016)	(0.018)	(0.015)	(0.020)
Lag_Approval	0.121***	0.342***	0.152***	0.013	0.230***	0.057	−0.068
	(0.019)	(0.035)	(0.029)	(0.021)	(0.034)	(0.033)	(0.037)
Agri Share	−0.027*	−0.032*	−0.042*	−0.054*	−0.050*	−0.036*	−0.047*
	(0.013)	(0.014)	(0.018)	(0.024)	(0.023)	(0.017)	(0.023)
Arable Land	0.725	−0.296	−0.291	−0.338	−0.120	−0.933	−0.324
	(1.472)	(0.998)	(1.129)	(1.056)	(0.783)	(0.801)	(0.742)
UPOV	0.660***	0.685***	0.703***	0.727***	0.403**	0.331*	0.366*
	(0.147)	(0.136)	(0.129)	(0.145)	(0.133)	(0.134)	(0.152)
Infrastructure	1.524	2.368	3.209	2.250	4.390*	4.399	4.066
	(2.081)	(1.958)	(2.203)	(2.373)	(2.176)	(2.466)	(2.223)
Observations	3574	3362	3150	2940	2733	2524	2314
R-squared	0.248	0.349	0.287	0.284	0.314	0.303	0.298

Note. The dependent variable is the number of GMO events approved, so coefficients are interpreted in unit terms. Horizons refer to the response years. Standard errors are in parentheses. Statistical significance is denoted by *** (1%), ** (5%), and * (10%) levels. Country fixed effect and commodity fixed effect are included.

## 7. Robustness checks: Droughts and drought-tolerance GMO approval

**Fig 6:**
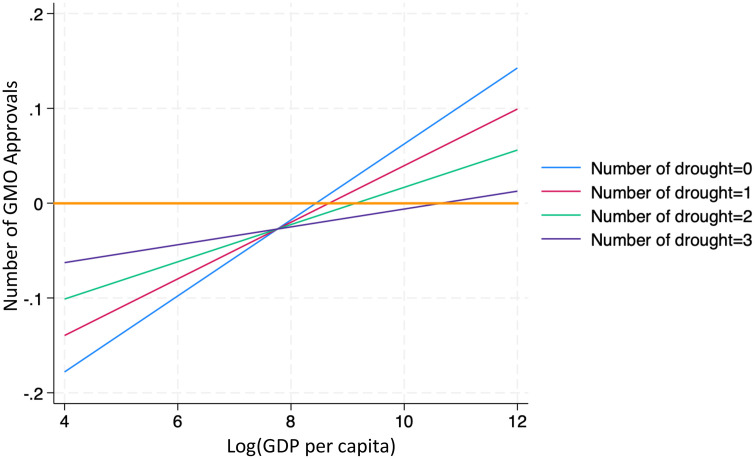
Marginal effects of the logarithm of GDP per capita on drought-related GMO approvals.

As a robustness check, this paper narrows its focus to drought-specific events to better isolate their impact on GMO approvals—particularly for drought-tolerant traits. Table 9 presents local projection estimates of the cumulative effects of drought shocks on approvals of drought-tolerant GMOs. Fig 6 visualizes the relationship between the logarithm of GDP per capita and the number of approvals seven years after the shock, holding the number of drought events constant.

**Table 9:** Impact of total droughts on drought-related GMO approvals.

	Horizon 1	Horizon 2	Horizon 3	Horizon 4	Horizon 5	Horizon 6	Horizon 7
	
Total Shock	0.011	0.019	−0.034	0.006	−0.007	0.003	0.053
	(0.034)	(0.020)	(0.041)	(0.040)	(0.047)	(0.028)	(0.045)
							
Log (GDP per capita)	0.007	0.025***	0.018**	0.010*	0.014**	0.037***	0.012
	(0.004)	(0.004)	(0.006)	(0.005)	(0.005)	(0.007)	(0.008)
							
Total Shock x Log (GDP per capita)	−0.002	−0.003	0.003	−0.001	0.001	−0.002	−0.007
	(0.005)	(0.003)	(0.005)	(0.006)	(0.006)	(0.004)	(0.006)
							
Lag_Approval	0.025***	−0.002	0.015**	0.022***	0.021***	0.000	0.021**
	(0.003)	(0.002)	(0.005)	(0.005)	(0.004)	(0.003)	(0.007)
							
Agri Share	0.000	0.002*	0.001	0.000	−0.001	0.001	−0.001
	(0.001)	(0.001)	(0.001)	(0.001)	(0.001)	(0.001)	(0.001)
							
Arable Land	−0.067	−0.084	−0.063	−0.033	0.022	−0.008	−0.011
	(0.038)	(0.048)	(0.051)	(0.044)	(0.054)	(0.050)	(0.075)
							
UPOV	−0.008*	0.004	−0.002	0.001	0.002	0.005	0.014*
	(0.003)	(0.004)	(0.004)	(0.004)	(0.005)	(0.005)	(0.007)
							
Infrastructure	−0.061	0.030	−0.014	0.055	0.286	0.326	0.123
	(0.116)	(0.145)	(0.163)	(0.126)	(0.175)	(0.220)	(0.229)
							
Observations	4308	4052	3796	3540	3284	3028	2772
R-squared	0.111	0.046	0.069	0.092	0.082	0.063	0.086

Note. The dependent variable is the number of GMO events approved, so coefficients are interpreted in unit terms. Horizons refer to the response years. Standard errors are in parentheses. Statistical significance is denoted by *** (1%), ** (5%), and * (10%) levels. Country fixed effect and commodity fixed effect are included.

The results reveal a similar pattern to previous findings: in lower-income countries, approvals of drought-tolerant GMOs remain negative even seven years after a drought. This suggests that despite the severe agricultural disruptions caused by droughts, countries with limited economic, regulatory, and technological capacity may hesitate to approve new GMO varieties. In contrast, higher-income countries—with more developed regulatory systems and access to scientific expertise—are more likely to respond by approving drought-tolerant GMOs to improve crop resilience and food security.

Therefore, this paper posits that the relationship between climate shocks and GMO approvals is shaped by a country’s comparative advantage in GMO technologies and its level of development. In countries with limited capacity or weak positioning in the global GMO market, protective or risk-averse policies may override the incentives to adopt GMOs in the face of climate shocks. Rather than accelerating innovation, climate stress may intensify regulatory hesitation or reinforce political resistance to GMOs. This dynamic can worsen food insecurity, especially in regions already vulnerable to climate-related agricultural disruptions. These results highlight the importance of designing adaptive GMO policies that reflect both national capabilities and the growing need for technological solutions in the face of increasing climate volatility.

## 8. Conclusion

This study investigates how climate shocks influence national decisions to approve GMOs. It finds that countries with a comparative disadvantage in GMO technologies are more likely to reduce GMO approvals following extreme climate events. Using both theoretical modeling and empirical analysis, the paper shows that climate shocks can weaken a country’s comparative advantage in GMO production and reduce the capital allocated to GMO-related technologies. Empirically, the paper uses the local projection method to estimate the cumulative effects of climate shocks on GMO approvals, controlling for country and commodity fixed effects as well as a set of relevant socioeconomic and institutional variables. The findings show that, on average, one additional climate shock reduces GMO approvals by 0.03 events four years later. Droughts and severe winter conditions emerge as the most disruptive climate events, with long-lasting negative effects on GMO approvals over a seven-year horizon.

This paper reveals the mechanism behind the impact of climate shocks on GMO approvals in two ways. First, GDP per capita is used as a proxy for a country’s capacity to invest in and implement GMO technologies. At lower income levels, countries tend to approve fewer GMOs—even several years after experiencing climate shocks. This pattern persists when focusing only on GMOs that have already been approved in other countries, and when the analysis is limited to drought shocks and drought-tolerant GMO traits. These results suggest that limited economic and technical capacity can constrain a country’s ability to adopt adaptive biotechnologies, even when faced with increasing climate risk.

Second, the paper generates a Revealed Comparative Advantage (RCA) measure to to separate countries into two groups—those with a comparative advantage in GMO production and those without. The results show that climate shocks have more persistent and negative effects on GMO approvals in countries with a comparative disadvantage. Specifically, four years after a shock, these countries experience a notable decline in approvals, while countries with a comparative advantage do not. This supports the view that protectionist responses and weak specialization can amplify regulatory hesitation, leading to stalled innovation and potential global disputes over GMO policies.

These findings carry important policy implications. As climate shocks become more frequent and severe, countries with limited technological capacity or weak comparative advantage in GMO production may fall further behind in adopting adaptive agricultural innovations. This delay not only undermines national food security efforts but may also deepen global disparities in resilience and productivity. To address this challenge, national policymakers should prioritize investments in agricultural research and development, strengthen biosafety regulatory frameworks, and enhance institutional capacity to evaluate and deploy GMO technologies effectively.

At the international level, cooperation is essential. Multilateral organizations, donors, and trade partners can help bridge these gaps by supporting harmonization of regulatory standards, facilitating access to pre-approved GMO events, and funding the technical infrastructure needed for risk assessment and monitoring. Encouraging transparent, science-based approval systems—particularly for GMO traits that have been vetted through international protocols—can reduce approval asymmetries and minimize delays in critical adaptive technologies. Additionally, trade agreements and global forums should consider the role of biotechnology in climate resilience and promote mechanisms that enable low- and middle-income countries to benefit from innovation diffusion. In sum, enhancing the institutional and regulatory foundations for GMO adoption in structurally disadvantaged regions is essential for building a more adaptive, inclusive, and secure global food system in the face of accelerating climate change.

## Supporting information

S1 Data Data R1. (CSV)
